# Regulatory Mechanisms of Vitellogenesis in Insects

**DOI:** 10.3389/fcell.2020.593613

**Published:** 2021-01-28

**Authors:** Zhongxia Wu, Libin Yang, Qiongjie He, Shutang Zhou

**Affiliations:** State Key Laboratory of Cotton Biology, Key Laboratory of Plant Stress Biology, School of Life Sciences, Henan University, Kaifeng, China

**Keywords:** vitellogenin, juvenile hormone, ecdysone, nutrition, insect reproduction

## Abstract

Vitellogenesis is pre-requisite to insect egg production and embryonic development after oviposition. During insect vitellogenesis, the yolk protein precursor vitellogenin (Vg) is mainly synthesized in the fat body, transported by the hemolymph through the intercellular spaces (known as patency) in the follicular epithelium to reach the membrane of maturing oocytes, and sequestered into the maturing oocytes via receptor-mediated endocytosis. Insect vitellogenesis is governed by two critical hormones, the sesquiterpenoid juvenile hormone (JH) and the ecdysteriod 20-hydroxyecdysone (20E). JH acts as the principal gonadotropic hormone to stimulate vitellogenesis in basal hemimetabolous and most holometabolous insects. 20E is critical for vitellogenesis in some hymenopterans, lepidopterans and dipterans. Furthermore, microRNA (miRNA) and nutritional (amino acid/Target of Rapamycin and insulin) pathways interplay with JH and 20E signaling cascades to control insect vitellogenesis. Revealing the regulatory mechanisms underlying insect vitellogenesis is critical for understanding insect reproduction and helpful for developing new strategies of insect pest control. Here, we outline the recent research progress in the molecular action of gonadotropic JH and 20E along with the role of miRNA and nutritional sensor in regulating insect vitellogenesis. We highlight the advancements in the regulatory mechanisms of insect vitellogenesis by the coordination of hormone, miRNA and nutritional signaling pathways.

## Introduction

A hallmark of female insect reproduction is vitellogenesis, a heterosynthetic process by which vitellogenin (Vg) is mostly synthesized in the fat body and deposited into developing oocytes. In addition to the fat body, Vg synthesis has been reported in follicle cells (Gilbert et al., 1998), nurse cells (Melo et al., 2000; Matsumoto et al., 2008) and hemocytes (Bai et al., 2015; Huo et al., 2018) in several insect species. Vg is critical for egg maturation during adulthood and embryonic growth after oviposition. In the honey bee *Apis mellifera*, Vg also protects the queen and long-lived worker bees from oxidative stress and further extends their life span (Seehuus et al., 2006; Corona et al., 2007; Ihle et al., 2015). Additionally, Vg plays a role in sensing fat body sugars and gustatory perception in the worker bees (Amdam et al., 2006; Wang et al., 2012, 2013). Furthermore, *A. mellifera* Vg acts as a pathogen recognition receptor and transports pathogen-derived molecules into the offspring to achieve trans-generational immunity (Garcia et al., 2010; Salmela et al., 2015; Zhang et al., 2015). In the small brown planthopper *Laodelphax striatellus*, Vg synthesized by hemocytes can facilitate the vertical transmission of rice stripe virus (Huo et al., 2018). The number of *Vg* genes among different insect species ranges from one to three in general, while the mosquito *Aedes aegypti* and the ant *Linepithema humile* possess up to five *Vg* genes (Corona et al., 2013) (Supplementary Table 1). The presence of multiple copies of *Vg* genes and multiple forms of Vg protein in insects is unclear. It is conceivable that two or more copies of *Vg* gene ensure the efficient production of yolk protein precursors required for the maturation of multiple eggs. The variation of *Vg* gene and protein in diverse insect species might reflect the evolutionary selection and the strategy of adaptation to environment (Garcia et al., 2010). Vg protein is a large glycolipophosphoprotein that is often oligomeric in their native state, with monomers consisting of two or more subunits (apoproteins). The molecular weights vary from 150 kDa to 200 kDa for large subunits and from 40 kDa to 65 kDa for small subunits, respectively (Lee et al., 2015). Vg protein sequences are evolutionarily conserved across insect orders except for yolk proteins (YPs) in Diptera. In the fruit fly *Drosophila melanogaster*, YPs form a different family of proteins to nurse maturing oocytes. Vg is generally composed of a lipoprotein N-terminal domain (LPD_N) for lipid binding, a 1943 domain with unknown function (DUF1943), and a von Willebrand factor type D domain (vWFD) in the C-terminus (Morandin et al., 2014). LPD_N has a conserved polyserine tract possessing consensus cleavage motifs (^R^/_K_XX^R^/_K_) (Tufail and Takeda, 2008). Vg proteins are phosphorylated at the polyserine tract, but the function of Vg phosphorylation remains elusive (Tufail and Takeda, 2008).

Cumulative studies have established that insect vitellogenesis is controlled by two classic hormones, the sesquiterpenoid juvenile hormone (JH) and the ecdysteroid 20-hydroxyecdysone (20E, an active form of ecdysone). Both JH and 20E can stimulate various aspects of vitellogenesis but vary across insect orders depending on reproductive traits (Wyatt and Davey, 1996; Raikhel et al., 2005; Roy et al., 2018; Santos et al., 2019; Song and Zhou, 2020). In evolutionarily primitive hemimetabolous insects such as the migratory locust *Locusta migratoria* and the German cockroach *Blattella germanica*, JH acts independently of 20E to stimulate vitellogenesis and oocyte maturation (Wyatt and Davey, 1996; Belles, 2004; Roy et al., 2018; Song and Zhou, 2020). In holometabolous coleopteran like the red flour beetle *Tribolium castaneum*, JH governs Vg synthesis in the fat body while 20E participates in ovarian growth and oocyte maturation (Parthasarathy et al., 2010a,b; Sheng et al., 2011). In many lepidopterans including the tobacco hornworm *Manduca sexta* and the cotton bollworm *Helicoverpa armigera*, in which Vg synthesis is initiated in adults, JH plays a principal role in vitellogenesis (Swevers and Iatrou, 2003; Telfer, 2009). However, in other lepidopterans such as the silkworm *Bombyx mori*, the silk moth *Hyalophora cecropia*, and the armyworm *Spodoptera frugiperda*, in which Vg is synthesized prior to adult ecdysis, 20E appears to have a primary role in vitellogenesis (Swevers and Iatrou, 2003; Telfer, 2009). In the mosquito *Ae. aegypti*, JH promotes fat body becoming competent for Vg synthesis, while 20E stimulates *Vg* expression and oocyte maturation after a blood meal (Raikhel et al., 2002; Shin et al., 2012). In *D. melanogaster*, 20E is responsible for the high rate of Vg synthesis in the fat body and JH controls Vg uptake into oocytes (Bownes, 1990; Carney and Bender, 2000; Berger and Dubrovsky, 2005).

Nutrients play a pivotal role in insect vitellogenesis. As nutritional sensors, the amino acid/Target of Rapamycin (AA/TOR) and insulin (or insulin-like peptide, ILP) are involved in biosynthesis of JH and 20E and crosstalk with JH and 20E pathways, which in turn regulate various aspects of insect vitellogenesis (Badisco et al., 2013; Hansen et al., 2014; Smykal and Raikhel, 2015; Roy et al., 2018). Furthermore, recent studies have revealed an essential role of microRNA (miRNA) in the regulation of insect vitellogenesis (Roy et al., 2018; Song and Zhou, 2020). Hence, this review covers the present status of our understanding in the regulatory mechanisms of insect vitellogenesis including Vg synthesis in the fat body and uptake in the ovary.

## Regulation of Vg Synthesis in the Fat Body

**Figure 1:**
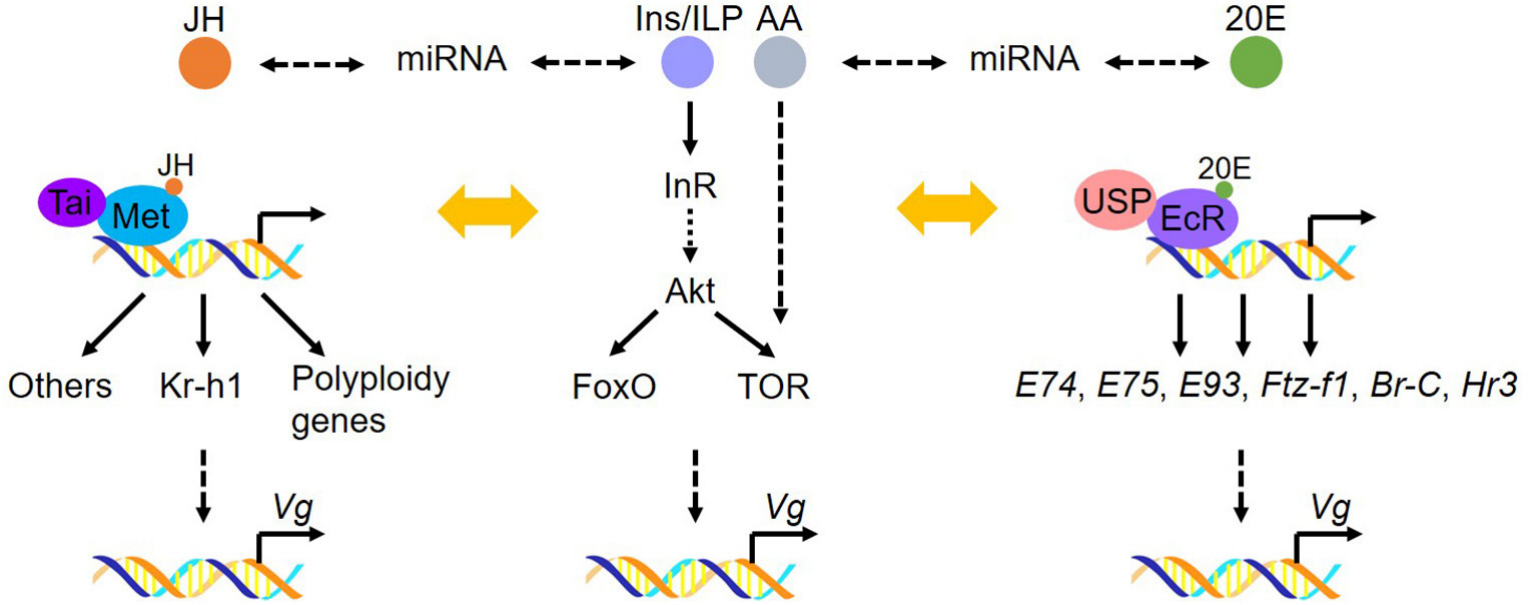
Molecular basis of Vg synthesis in the fat body. JH receptor complex Met/Tai directly regulates the transcription of target genes encoding *Kr-h1* and the polyploidy genes (including DNA replication and cell cycle genes). 20E receptor complex EcR/USP directly activates the expression of 20E-responsive genes. Nutritional signaling is involved in Vg production in the fat body by interplay with JH or 20E. In addition, miRNA participates in JH- or 20E-dominant Vg synthesis in the fat body. These factors directly or indirectly regulate *Vg* transcription and vitellogenesis.

Insect fat body, analogous to the vertebrate liver and adipose tissue, is the major source for Vg synthesis. Recently, research advancements have been achieved in the molecular mechanisms of Vg synthesis regulated by JH, 20E, miRNA and nutritional pathways. For the regulatory mechanisms of 20E, AA/TOR and peptides in insect vitellogenesis and oogenesis, we refer readers to three recent reviews (Roy et al., 2018; Lenaerts et al., 2019; Swevers, 2019). Here, we outline the recent accomplishments in regulating Vg synthesis in the fat body, with the emphasis on the role of JH, miRNA and ILP pathways (Figure 1).

### JH Action on Vg Synthesis

The molecular action of JH relies on its intracellular receptor Methoprene-tolerant (Met), a member of basic helix-loop-helix Per-Arnt-Sim (bHLH-PAS) transcription factor family. JH induces the heterodimerization of Met with another bHLH-PAS protein, Taiman (Tai) to form an active JH-receptor complex (Jindra et al., 2013). The JH-Met/Tai receptor complex consequently activates the transcription of JH-responsive genes (Charles et al., 2011; Li et al., 2011; Kayukawa et al., 2012; Guo et al., 2014; Jindra et al., 2015; Wu et al., 2016, 2018; Wang Z. et al., 2017). In adult insects, JH requires the Met/Tai complex to achieve its previtellogenic and vitellogenic effects on fat body competency and Vg synthesis (Zou et al., 2013; Guo et al., 2014; Gujar and Palli, 2016; Wang Z. et al., 2017). RNAi-mediated knockdown of *Met* resulted in significant reduction of *Vg* expression level accompanied by arrested oocyte maturation and blocked egg production in a variety of investigated insects including *T. castaneum, L. migratoria, H. armigera*, the linden bug *Pyrrhocoris apterus*, the brown planthopper *Nilaparvata lugens*, the oriental fruit fly *Bactrocera dorsalis*, and the white-backed planthopper *Sogatella furcifera* (Parthasarathy et al., 2010b; Shpigler et al., 2014; Smykal et al., 2014; Song et al., 2014; Lin et al., 2015; Ma et al., 2018; Yue et al., 2018; Hu et al., 2019). In *L. migratoria, P. apterus*, and *Ae. aegypti*, depletion of *Tai* or its orthologs *FISC* and *p160/SRC* led to significantly declined levels of *Vg* transcripts along with blocked oocyte development (Smykal et al., 2014; Wang Z. et al., 2017). *Krüppel-homolog 1* (*Kr-h1*), a primary JH early-response gene that codes for a zinc finger transcription factor, transduces JH signaling in regulating adult reproduction (Kayukawa et al., 2012; Shin et al., 2012; Song et al., 2014). The role of Kr-h1 in insect vitellogenesis varies among insect species. In *L. migratoria, B. dorsalis*, and the rice stem borer *Chilo suppressalis*, Kr-h1 mediates JH action to promote vitellogenesis and oocyte maturation (Song et al., 2014; Yue et al., 2018; Tang et al., 2020). In *Ae. aegypti*, Kr-h1 transduces JH signaling as both activator and repressor to regulate the expression of JH-responsive genes involved in previtellogenic development, consequently regulating egg production after a blood meal (Shin et al., 2012; Zou et al., 2013; Ojani et al., 2018). In *T. castaneum*, depletion of *Met* caused more than 80% decrease of *Vg* mRNA levels, whereas *Kr-h1* knockdown resulted in about 30% reduction of *Vg* transcripts (Parthasarathy et al., 2010b). In *P. apterus*, knockdown of *Met* or *Tai* but not *Kr-h1* suppressed Vg synthesis in the fat body (Smykal et al., 2014). In the common bed bug *Cimex lectularius, Kr-h1* knockdown appeared to have no significant impact on vitellogenesis (Gujar and Palli, 2016).

*L. migratoria*, a hemimetabolous species bearing the panoistic ovary and synchronously matured oocytes, has been a long-standing model for studying JH-dependent female reproduction (Wyatt and Davey, 1996; Roy et al., 2018). In adult females of *L. migratoria*, JH induces Vg synthesis in the fat body, initiates intercellular space (patency) in the follicular epithelium, and facilitates the uptake of Vg into developing oocytes (Wyatt and Davey, 1996; Song and Zhou, 2020). During the first gonadotrophic cycle, locust fat body undergoes endocycle to produce up to 32C (chromatin copies per cell) polyploid cells required for massive Vg synthesis (Guo et al., 2014). RNA-seq-based gene expression profiling followed by qRT-PCR validation demonstrated that JH upregulated the expression of 16 genes related to DNA replication and 13 genes involved in cell cycle progression, suggesting that JH plays a pivotal role in the onset and progression of polyploidy by promoting endocycle in the fat body (Guo et al., 2014). Of these 29 genes, chromosome maintenance genes 3, 4, 7 (*Mcm3, Mcm4*, and *Mcm7*), *cell-division-cycle 6* (*Cdc6*), *cyclin-dependent kinase 6* (*Cdk6*) and *adenovirus E2 factor-1* (*E2f1*) expressed in response to both JH and Met (Guo et al., 2014). Further studies revealed that the JH-Met/Tai receptor complex bound to the consensus sequences with E-box or E-box-like motifs in the promoters of *Mcm4, Mcm7, Cdc6, Cdk6*, and *E2f1* and activated their transcription. Knockdown of *Mcm4, Mcm7, Cdc6, Cdk6*, or *E2f1* in vitellogenic female locusts resulted in significant reduction in fat body cell ploidy, accompanied by markedly reduced *Vg* expression levels, arrested oocyte maturation and impaired egg production (Guo et al., 2014; Wu et al., 2016, 2018). Interestingly, depletion of *Met* had no significant effect on other genes upregulated by JH (Guo et al., 2014), suggesting the involvement of other signaling cascades that interplay with JH. Next, Wu et al. (2020) demonstrated that JH stimulated FoxO dephosphorylation through leucine carboxyl methyltransferase 1 (LCMT1)-mediated activation of protein phosphatase 2A (PP2A). JH-LCMT1-PP2A axis-triggered FoxO dephosphorylation facilitated FoxO translocation in nuclei and activated the transcription of *cell-division-cycle 2* (*Cdc2)* and *origin-recognition-complex subunit 5* (*Orc5*). Knockdown of *LCMT1, PP2A, FoxO, Cdc2*, or *Orc5* in vitellogenic female locusts resulted in lower ploidy and significantly reduced *Vg* expression in the fat body as well as arrested oocyte maturation and suppressed egg development (Wu et al., 2020). Collectively, JH acts via its receptor complex Met/Tai to induce the expression of *Mcm4*/*7, Cdc6, Cdk6*, and *E2f1*, and through LCMT1-PP2A-FoxO axis to trigger the expression of *Cdc2* and *Orc5* (Guo et al., 2014; Wu et al., 2016, 2018, 2020). These two signaling pathways coordinate to promote fat body cell polyploidization for large-scale Vg synthesis, which meets the requirement of synchronous maturation of multiple eggs in *L. migratoria*.

*Ae. aegypti* serves as an ideal model for studying JH-dependent posteclosion or previtellogenic development. Besides the JH-Met/Tai complex, JH induces the dimerization of Met with Cycle to upregulate gene transcription for previtellogenic development in *Ae. aegypti* (Shin et al., 2012). JH acts via Met to induce the expression of *Regulator of Ribosome Synthesis 1* (*RRS1*) and *Ribosomal protein L32* (*RpL32*), consequently enhancing ribosomal biogenesis in the fat body essential for massive Vg synthesis (Wang J. L. et al., 2017). Saha et al. (2019) reported that JH induced the expression of gene coding for Hairy which dimerizes with Groucho to form a transcriptional repressor complex mediating the repressive function of JH. Interestingly, Hairy and Kr-h1 acted synergistically in the JH/Met gene repression hierarchy during previtellogenic development of *Ae. aegypti* (Saha et al., 2019). JH exerts both genomic and non-genomic action. For non-genomic action, JH acts via the RTK-PLC-IP3-CaMKII signaling cascade to trigger Met phosphorylation that enhances its transcriptional activity in previtellogenic development of *Ae. aegypti* (Liu et al., 2015; Ojani et al., 2016). Moreover, JH triggers the RTK-PI3K-Akt signaling pathway to stimulate the phosphorylation of serine/arginine-rich (pre-mRNA) splicing factor (SRSF), which induces the alternative splicing of Tai to produce Tai-A and Tai-B isoforms (Liu et al., 2018). These two isoforms could potentiate the transcriptional activity of 20E-receptor in the fat body of adult female mosquitoes, consequently promoting blood meal-induced vitellogenesis and egg production (Liu et al., 2018).

### Nutritional Control of Vg Synthesis

AA/TOR pathway has been demonstrated to play pivotal roles in activating Vg synthesis in divergent orders of insects (Sancak et al., 2008; Carpenter et al., 2012; Koyama et al., 2013; Lu et al., 2016). Suppression of TOR activity by rapamycin treatment or RNA-mediated knockdown inhibited *Vg* expression in *Ae. aegypti* and *N. lugens* (Hansen et al., 2004; Lu et al., 2016). Moreover, TOR tends to have a positive effect on JH biosynthesis and vitellogenesis. In *T. castaneum, B. germanica*, and the American cockroach *Periplaneta americana*, TOR and ILP signaling could enhance the expression of *JH methyltransferase* (*JHAMT*), *Met* and *Kr-h1*, further stimulating *Vg* expression and oocyte maturation (Parthasarathy and Palli, 2011; Abrisqueta et al., 2014; Zhu et al., 2020). Moreover, insulin/TOR signaling affects 20E synthesis and secretion as well, further associates with vitellogenesis and egg production in *D. melanogaster* and *Ae. aegypti* (Tu et al., 2002; Dhara et al., 2013).

FoxO plays a vital role in mediating the crosstalk between insulin and JH signaling pathways to coordinate insect vitellogenesis (Koyama et al., 2013; Smykal and Raikhel, 2015; Roy et al., 2018; Santos et al., 2019). In *B. germanica*, depletion of insulin receptor gene (*InR*) or *FoxO* in fed adult females resulted in markedly reduced *Vg* expression along with impaired oocyte maturation (Abrisqueta et al., 2014). Similarly, *FoxO* knockdown led to reduction of *Vg* transcript levels in fed adult females of *T. castaneum, Ae. aegypti, L. migratoria* and the soybean pod borer *Maruca vitrata* (Hansen et al., 2007; Parthasarathy and Palli, 2011; Abrisqueta et al., 2014; Al Baki et al., 2019; Wu et al., 2020). However, silencing of *FoxO* in the starved adult females of *B. germanica* caused significant increase of *Vg* expression due to elevated biosynthesis of JH (Suren-Castillo et al., 2012; Abrisqueta et al., 2014). In *B. mori*, FoxO suppresses the expression of JH degradation genes *JHE, JHDK*, and *JHEH* to protect JH from degradation (Zeng et al., 2017). Interestingly, FoxO binds to *Vg2* gene promoter and inhibits its transcription in *T. castaneum*. After adult emergence, JH induces *ILP* expression and FoxO phosphorylation, which in turn releases FoxO binding and activates *Vg2* transcription (Sheng et al., 2011).

### miRNA Regulation of Vg Synthesis

As fine-tuners, miRNAs generally downregulate their target genes by translation inhibition or mRNA degradation in a spatiotemporal manner. Nevertheless, miRNAs can bind the 5′-UTR or CDS of target mRNAs to upregulate gene expression (Vasudevan et al., 2007; Zhou et al., 2009; Yang et al., 2014; He et al., 2016). The role of miRNAs in oogenesis has been extensively studied in *D. melanogaster* (Asgari, 2013; Shcherbata, 2019; Song and Zhou, 2020). Recently, miRNA functions in vitellogenesis and oocyte maturation have been explored in non-model insects like *Ae. aegypti* and *L. migratoria* (Lucas et al., 2015b; Roy et al., 2018; Song and Zhou, 2020). As two previous reviews have covered miRNA regulation in insect reproduction (Roy et al., 2018; Song and Zhou, 2020), we update the recent advances in the role of miRNAs in insect vitellogenesis.

Dicer 1 and Argonaute1 (Ago1) are two key enzymes involved in miRNA biogenesis and functioning. Depletion of *Dicer 1* or *Ago1* in *D. melanogaster, B. germanica, L. migratoria*, and *N. Lugens* led to defective phenotypes of female reproduction including decreased *Vg* expression and impaired oocyte maturation (Jin and Xie, 2007; Azzam et al., 2012; Tanaka and Piulachs, 2012; Song et al., 2013; Zhang et al., 2013). In adult female locusts, JH upregulated 59 miRNAs and downregulated 23 miRNAs (Song et al., 2013). Of JH-downregulated miRNAs, let-7 and miR-278 bound to *Kr-h1* coding sequence and repressed its expression. Application of let-7 and miR-278 agomiRs resulted in significantly reduced *Kr-h1* expression along with markedly decreased abundance of Vg protein and blocked oocyte maturation (Song et al., 2018). Intriguingly, JH titer increases from the previtellogenic stage to vitellogenic phase, whereas let-7 and miR-278 have the opposite tendency in abundance (Song et al., 2018; Guo et al., 2019). The increased JH titer and declined abundance of let-7 and miR-278, therefore, ensure high levels of Kr-h1 required for vitellogenesis. miR-2/13/71 is clustered miRNAs downregulated by JH in *L. migratoria* (Song et al., 2019). miR-2/13/71 bound to the coding sequence of *Notch* mRNA to inhibit its expression. Injection of miR-2/13/71 agomiRs in adult female locusts also led to reduced *Vg* transcripts and arrested oocyte maturation (Song et al., 2019). Likewise, the increased JH titer and declined abundance of miR-2/13/71 in vitellogenic female locusts contribute to high levels of Notch for successful vitellogenesis and egg development (Song et al., 2019).

In *Ae. aegypti*, lineage-specific miR-1890 targets the 3′UTR of JH-regulated *Chymotrypsin-like serine protease* (*JHA15*). After a blood meal, 20E suppresses miR-1890 expression to prevent adult females from impaired blood digestion in the midgut and Vg synthesis in the fat body (Lucas et al., 2015b). Disruption of miR-275, miR-1174 or miR-8 also caused defects in blood digestion, vitellogenesis and egg production in the vitellogenic females of *Ae. aegypti* (Bryant et al., 2010; Liu et al., 2014; Lucas et al., 2015a). Moreover, miR-989, most abundant in the fat body and ovary of *Ae. aegypti*, directly targets *Vg* gene during female reproduction (Zhang et al., 2017). In the malaria mosquito *Anopheles gambiae*, miR-309 antagomiR treatment caused impaired vitellogenesis and egg production (Fu et al., 2017). In *N. lugens*, miR-4868b inhibits the expression of *Glutamine synthase* gene. miR-4868b antagomiR treatment resulted in significant decrease of Vg protein levels along with impeded egg development and reduced fecundity (Fu et al., 2015).

### Post-translational Regulation of Synthesized Vg

Nascently synthesized Vg in the fat body is properly folded in the endoplasmic reticulum (ER) and assembled into the secretory vesicles via membrane fusion with the Golgi apparatus. The 78-kDa Glucose-regulated protein (Grp78), a heat shock protein 70 kDa family member is one of the most abundant chaperones in fat body ER of *L. migratoria* (Luo et al., 2017). During locust vitellogenesis, JH stimulates two *Grp78* genes, *Grp78-1* and *Grp78-2* via differential signaling pathways, which together accelerate the production of Grp78 chaperone. Grp78 facilitates the proper folding of massively synthesized Vg and avoid ER stress in the fat body (Luo et al., 2017). During the intracellular transportation, Vg stability is threatened by a variety of proteases but protected by protease inhibitors, of which the Kazal-type protease inhibitor is a common subfamily (Van Hoef et al., 2013). Guo et al. (2019) demonstrated that Greglinwas the predominant Kazal-type protease inhibitor in the fat body and ovary of vitellogenic females of *L. migratoria*. During locust vitellogenesis, JH induced high levels of *Greglin* expression to protect Vg from proteolysis (Guo et al., 2019). During the termination of *B. mori* embryonic diapause under low temperature, extracellular signal-regulated kinase (ERK)/mitogen-activated protein kinase (MAPK) signaling is activated in the yolk cells (Iwata et al., 2005). Thereafter, ERK/MAPK increases sorbitol and 20E metabolism by regulating the transcription of downstream genes. The elevated sorbitol-glycogen conversion and 20E secretion promote embryonic development, yolk-cell dispersion as well as yolk protein degradation (Fujiwara et al., 2006).

## Regulation of Vg Uptake by Ovaries

**Figure 2:**
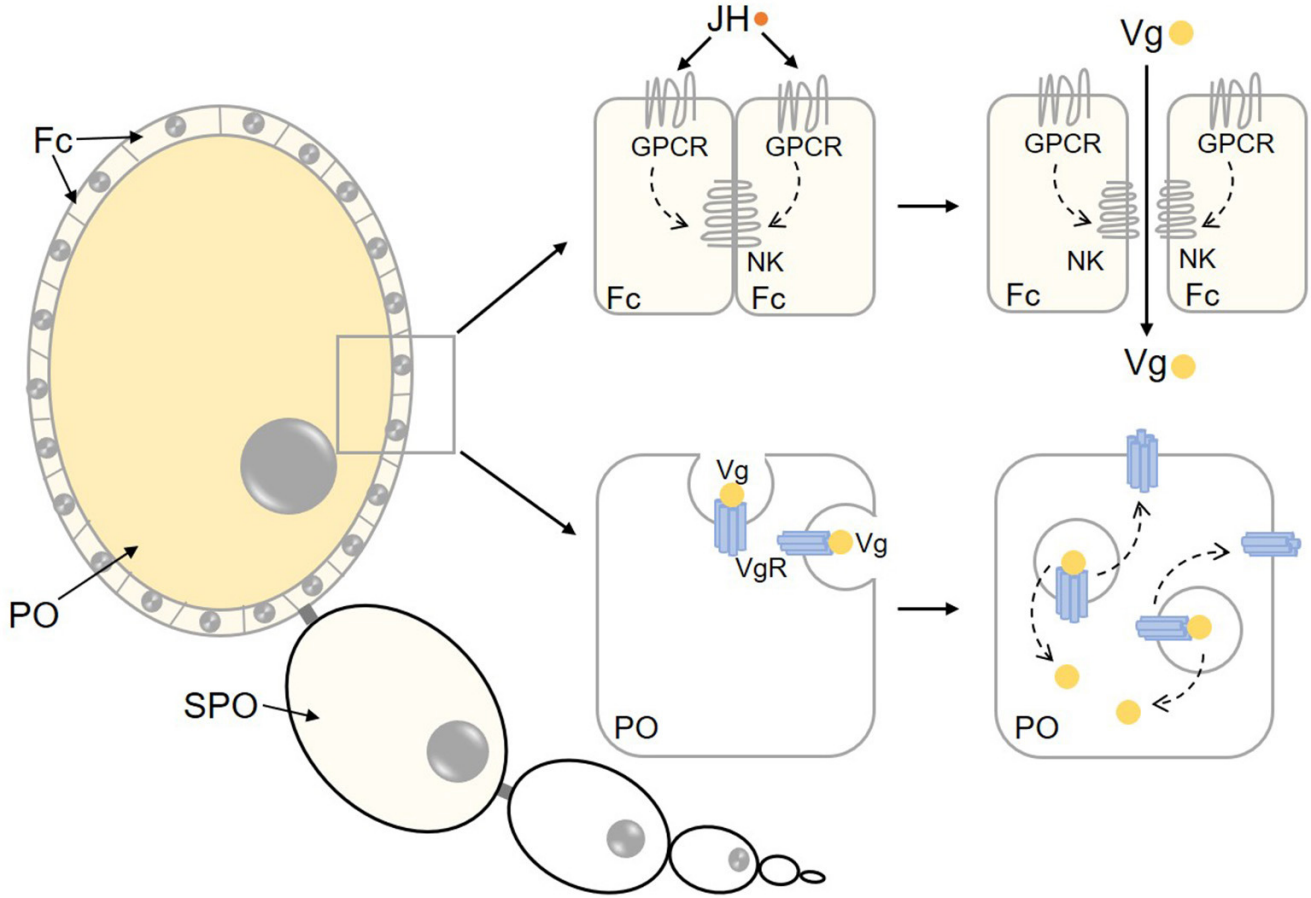
Regulation of Vg uptake by maturing oocytes. Prior to Vg uptake by maturing oocytes, Vg passes through patency among follicle cells, which is regualted by JH action on GPCR and Na^+^/K^+^-ATPase. Vg is internalized by surrounding oocytes through VgR-mediated endocytosis. Afterwards, Vg is unbound with VgR, then VgR is recycled to the membrane of the oocyte. NK, Na^+^/K^+^-ATPase; Fc, follicle cells; PO, primary oocyte; SPO, sub-primary oocyte.

With the help of hemolymph circulation, Vg is transported into the ovary and absorbed by maturing oocytes. Oocytes are enclosed by the follicle epithelium, which determines the size and shape of oocytes and initiates the intercellular spaces (patency) (Wyatt and Davey, 1996; Raikhel et al., 2005). Vg is transported through patency to the surface of competent oocytes and then internalized via Vg receptor (VgR)-mediated endocytosis (Figure 2). JH plays a crucial role in Vg uptake by ovaries in a variety of insects. In adult females of *H. armigera*, depletion of *Met* severely suppressed *Vg* and *VgR* transcription, disrupted yolk protein uptake into oocytes, and eventually led to reduced fecundity (Ma et al., 2018). In *L. migratoria*, knockdown of *Met* or *Kr-h1* caused markedly smaller follicle cells and malfunctional patency (Song et al., 2014). In *B. germanica*, the JH-dependent expression of *SPARC* (*secreted protein, acidic and rich in cysteine*) gene, coding for a calcium-binding glycoprotein that forms part of the extracellular membranes is critical for follicle cell morphological changes and patency induction that facilitate Vg uptake by developing oocytes (Irles et al., 2017).

### Role of Na^+^/K^+^-ATPase in Patency Initiation

Na^+^/K^+^-ATPase is a heterodimer protein that consists of α- and β-subunits (Lingrel and Kuntzweiler, 1994). The α-subunit, which has 10 transmembrane loops and large cytoplasmic regions, plays a chief function in catalytic action because of its ATP binding site and phosphorylation (Fambrough et al., 1994; Lingrel and Kuntzweiler, 1994). The β-subunit, also known as Nervana, is a nervous system-specific glycoprotein antigen in the adult head of *D. melanogaster*. The β-subunit contains a single transmembrane, a small N-terminal cytoplasmic domain and a large C-terminal extracellular region (Kaplan, 2002). In *B. germanica*, the orthologs of *D. melanogaster* Nervana 1 and Nervana 2 are crucial for oogenesis (Irles et al., 2013). Early study on the kissing bug *Rhodnius prolixus* has demonstrated that JH-stimulated patency is inhibited by application of ouabain, a specific inhibitor of Na^+^/K^+^-ATPase (Sevala and Davey, 1989), indicating the involvement of Na^+^/K^+^-ATPase in the regulation of JH-dependent patency initiation and Vg uptake. Subsequently, the role of Na^+^/K^+^-ATPase in patency initiation has been reported in *L. migratoria*, the beetle *Tenebrio molitor* and the moth *Heliothis virescens* (Sevala and Davey, 1993; Pszczolkowski et al., 2005). In *L. migratoria*, RNAi-mediated knockdown of *Na*^+^*/K*^+^*-ATPase* gene, pharmaceutical inhibition of Na^+^/K^+^-ATPase phosphorylation or suppression of Na^+^/K^+^-ATPase activity by inhibitor treatment resulted in the loss of patency along with blocked Vg uptake and impaired egg development (Jing et al., 2018).

As patency is initiated by JH, efforts have been made to elucidate the regulatory mechanisms of Na^+^/K^+^-ATPase activation by JH. Pharmaceutical approaches with protein kinase C (PKC) activator in *R. prolixus* have shown that PKC activation is required for the induction of JH-dependent Na^+^/K^+^-ATPase activity (Sevala and Davey, 1989). JH also evokes Na^+^/K^+^-ATPase-activated patency in *H. virescens* via the signaling pathways including protein kinase A (PKA) and PKC (Pszczolkowski et al., 2005, 2008). Jing et al. (2018) demonstrated that JH triggered a signaling cascade including G protein-coupled receptor (GPCR), receptor tyrosine kinase (RTK), phospholipase C (PLC), inositol trisphosphate (IP3) and PKC to phosphorylate Na^+^/K^+^-ATPase α-subunit at serine residue 8, leading to activation of Na^+^/K^+^-ATPase. Activated Na^+^/K^+^-ATPase is likely to change the ionic balance of follicle cells and cause cell shrinkage, consequently initiating patency for Vg transportation to the surface of maturing oocytes (Jing et al., 2018). However, the specific GPCR or RTK involved in GPCR/RTK-PLC-IP3-PKC signaling cascade remains to be identified. Bai and Palli (2016) reported that Dopamine D2-like receptor (Dop2R) controls Vg uptake by maturing oocytes in *T. castaneum* (Bai and Palli, 2016). In *L. migratoria*, 22 GPCRs were identified in the ovarian transcriptome of vitellogenic females. RNAi screening revealed that Orphan receptor-A (OR-A1 and OR-A2), C-type muscarinic acetylcholine receptor (mAChR-C) and Cirl-like (CirlL) played key roles in ovarian Vg transportation and uptake (Zheng et al., 2020).

### VgR-mediated Vg Endocytosis

Insect VgR belongs to the low-density lipoprotein receptor (LDLR) family (Raikhel and Dhadialla, 1992; Schonbaum et al., 1995; Tufail and Takeda, 2009). Upon binding of Vg to VgR, the Vg/VgR complex clusters in clathrin-coated pits, invaginates into the cytoplasm and pinches off to form intracellular coated vesicles. After transformed into the endosome or transitional yolk body, Vg and VgR are dissociated by ATP-dependent acidification. Subsequently, VgR is recycled back to oocyte membrane surface while Vg is crystallized and stored as vitellin (Vn) in yolk bodies for future embryonic development (Sappington and Raikhel, 1998; Raikhel et al., 2002; Snigirevskaya and Raikhel, 2005; Mitchell and Pérez De León, 2019). In the oriental fruit fly *Bactrocera dorsalis*, depletion of *VgR* blocked Vg uptake and egg development (Cong et al., 2015). Also, knockdown of *VgR* in *N. lugens* and *S. furcifera* caused accumulation of Vg in the hemolymph accompanied by arrested oocyte maturation (Lu et al., 2015; Hu et al., 2019). *VgR* expression is hormonally regulated during reproductive development. In the fire ant *Solenopsis invicta*, JH treatment of cultured ovaries significantly increased *VgR* expression (Chen et al., 2004). Analogously, JH treatment induced *VgR* expression in adult females of *N. lugens* (Lu et al., 2015). Furthermore, JH upregulates *VgR* expression by Met-Kr-h1 pathway in the cabbage beetle *Colaphellus bowringi* (Liu et al., 2019). Interestingly, in *P. americana* whose vitellogenesis is dependent upon JH, the expression of *Vg* and *VgR* is inhibited by additional 20E treatment (Kamruzzaman et al., 2020). Similarly, knockdown of the Halloween genes coding for a group of enzymes involved in 20E synthesis remarkably reduced the levels of *Vg* and *VgR* expression, leading to defective egg development of the diamondback moth *Plutella xylostella* (Peng et al., 2019). In *M. vitrata* and the beet armyworm *Spodoptera exigua*, the expression of *VgR* is regulated by ILP/TOR signaling pathway (Zhao et al., 2018; Al Baki et al., 2019). Notably, two miRNAs, miR-2739 and miR-167 bind directly to the 3′UTR of *B. mori VgR* mRNA and coordinately inhibit *VgR* expression. Overexpression of either miR-2739 or miR-167 using the piggyBac system blocked Vg deposition into oocytes, whereas application of miR-2739 or miR-167 antagomiR resulted in increase of VgR levels and uptake of Vg by oocytes (Chen et al., 2020).

In addition to mediating Vg internalization, VgR is shown to play an important role in vertical transmission of pathogenic microbes and *Wolbachia* symbionts (Guo et al., 2018; Huo et al., 2019; Mitchell and Pérez De León, 2019). In *L. striatellus*, Vg is hitchhiked by the rice stripe virus (RSV), which enters the developing oocytes through VgR-mediated endocytosis (Huo et al., 2018, 2019; He et al., 2019). Also in *L. striatellus, Wolbachia* symbionts acquire maternal transmission through the Vg-VgR axis (Guo et al., 2018). It has been demonstrated that a series of small guanosine triphosphatases (GTPase), whose activities are at peak during vitellogenesis, are involved in the process of Vg vesicular formation, budding, docking and fusion. In *P*. *americana*, GTPase activity is required for the intracellular trafficking of Vg in the ovary (Elmogy et al., 2014). In *N. lugens*, depletion of *Ran*, a member of GTPase family, brough about markedly reduced *Vg* expression and decreased fecundity (Li et al., 2015).

## Conclusions and Prospects

Vitellogenesis is one of the most emblematic processes in insect reproduction. Over the past decades, accumulative studies have advanced our understanding of how insect vitellogenesis is governed by hormonal and nutritional pathways. 20E acts through its receptor complex comprised of Ecdysone Receptor (EcR) and Ultraspiracle (USP), leading to transcriptional activation of 20E-response genes including *ecdysone-induced proteins 74* (*E74*), 75 (*E75*), *93F* (*E93*), *Broad-Complex* (*Br-C*), and *Ftz-f1*. The 20E pathway appears to be evolutionarily conserved in vitellogenesis and oogenesis across insect orders (Raikhel et al., 2005; Swevers, 2019). Most recently, relative more research progress has been made on the action of JH, nutrition and miRNA pathways on vitellogenesis in diverse insect species such as *L. migratoria, Ae. aegypti, T. castaneum*, and *B. germanica* (Roy et al., 2018; Santos et al., 2019; Song and Zhou, 2020). JH acts through its receptor complex or interplays with ILP pathway to stimulate fat body cells undergoing endocycle for polyploidization, which accelerates massive Vg synthesis to meet the requirement for synchronous maturation of multiple eggs in *L. migratoria* (Guo et al., 2014; Wu et al., 2016, 2018, 2020). Moreover, JH acts via Met and intracellular signaling cascades to promote fat body competency ready for Vg synthesis after a blood meal in *Ae. aegypti* (Shin et al., 2012; Ojani et al., 2016; Liu et al., 2018; Saha et al., 2019). The genomic action of JH has been extensively studied since the establishment of Met/Tai as the nuclear JH receptor and the identification of JH-responsive genes. Nevertheless, most studies on JH actions have been conducted in insect molting and metamorphosis. The molecular mechanisms of JH regulation in insect vitellogenesis and oogenesis are largely obscure. The membrane receptor of JH remains to be identified. The roles of JH and 20E in insect reproduction vary across insect orders and even among genera, it is of interest to unveil the evolutionary aspects of hormonal regulation in insect vitellogenesis. In addition, AA/TOR and ILP pathways play pivotal roles in insect vitellogenesis by way of direct regulation or interaction with JH signaling cascade (Parthasarathy and Palli, 2011; Abrisqueta et al., 2014; Roy et al., 2018; Zhu et al., 2020). Moreover, an increasing body of evidence supports the importance of miRNA regulation in insect vitellogenesis. Likewise, miRNAs can interact with JH pathway and modulate JH production, consequently regulating insect vitellogenesis (Roy et al., 2018; Song and Zhou, 2020). Interestingly, JH, miRNAs and cognate genes constitute the regulatory loop and coordinately control Vg synthesis (Song et al., 2018, 2019). Therefore, the molecular basis of JH, miRNA and nutritional pathway crosstalk in regulating insect Vg synthesis and uptake requires further exploration. Furthermore, the involvement of other epigenetic and posttranscriptional regulators in insect vitellogenesis need to be dissected. Currently, studies on Vg secretion from the fat body into the hemolymph and Vg uptake in the ovary are limited. Future discovery of new players in JH-dependent patency initiation in the follicular epithelium and VgR-mediated endocytosis in the oocytes will help construct the regulatory networks controlling insect vitellogenesis and egg production.

## Author Contributions

ZW, LY, and QH collected the references. ZW, LY and SZ wrote the manuscript. All authors contributed to the article and approved the submitted version.

## Conflict of Interest

The authors declare that the research was conducted in the absence of any commercial or financial relationships that could be construed as a potential conflict of interest.

## Supplementary Material

Supplementary Table 1 Identification of Vg genes in insects.
